# Integrated omics analysis of the cellulose co-degradation network of *Chaetomium thermophilum*

**DOI:** 10.1186/s13068-026-02741-x

**Published:** 2026-01-24

**Authors:** Xinran Yu, Su Ma, Xiuyun Wu, Lushan Wang

**Affiliations:** State Key Laboratory of Microbial Technology, Shandong University, Qingdao, 266237 China

**Keywords:** *Chaetomium thermophilum*, Cellulose degradation, Transcription factor Clr-2, Cellulose degradation enzymes, Co-degradation network, Gluconic acid metabolism

## Abstract

**Background:**

Efficient degradation of cellulose is a key bottleneck in the industrialization of biofuels. While fungi achieve substrate conversion through precise regulation of cellulase systems, the systematic mechanisms underlying efficient degradation (encompassing gene transcription, extracellular protein cooperation, and product metabolism) remain unclear in specific fungi, especially thermophilic fungi critical for industrial production.

**Results:**

(1) *C. thermophilum* did not induce cellulases under cellobiose, while microcrystalline cellulose (MCC) strongly activated degradation. *Ct*Clr-2 acts as a core transcription factor, directly driving the co-expression of key genes including LPMOs, CDH, and CBH; its deletion reduces MCC degradation efficiency by 30%. (2) Enzyme secretion may follow a three-stage cascade pattern (CBH1-A → LPMOs/CDH-1 → CBH1/2-B), where the selective secretion and temporal synergy of oxidases and hydrolase increase the reducing sugar yield by 60.6%. (3) The sugar acid metabolic network may enable efficient utilization of degradation products and potentially help maintain extracellular pH.

**Conclusions:**

This study reveals the efficient "transcriptional regulation-enzyme secretion adaptation" synergistic mechanism in *C. thermophilum*. *Ct*Clr-2 coordinates key genes, and staged enzyme secretion optimizes synergy, while sugar acid metabolism ensures homeostasis. These insights advance thermophilic cellulolysis understanding and provide targets for engineering industrial strains through synthetic biology (for example, enhancing enzyme yield and optimizing degradation efficiency), aiding cost reduction in biofuel production.

**Graphical Abstract:**

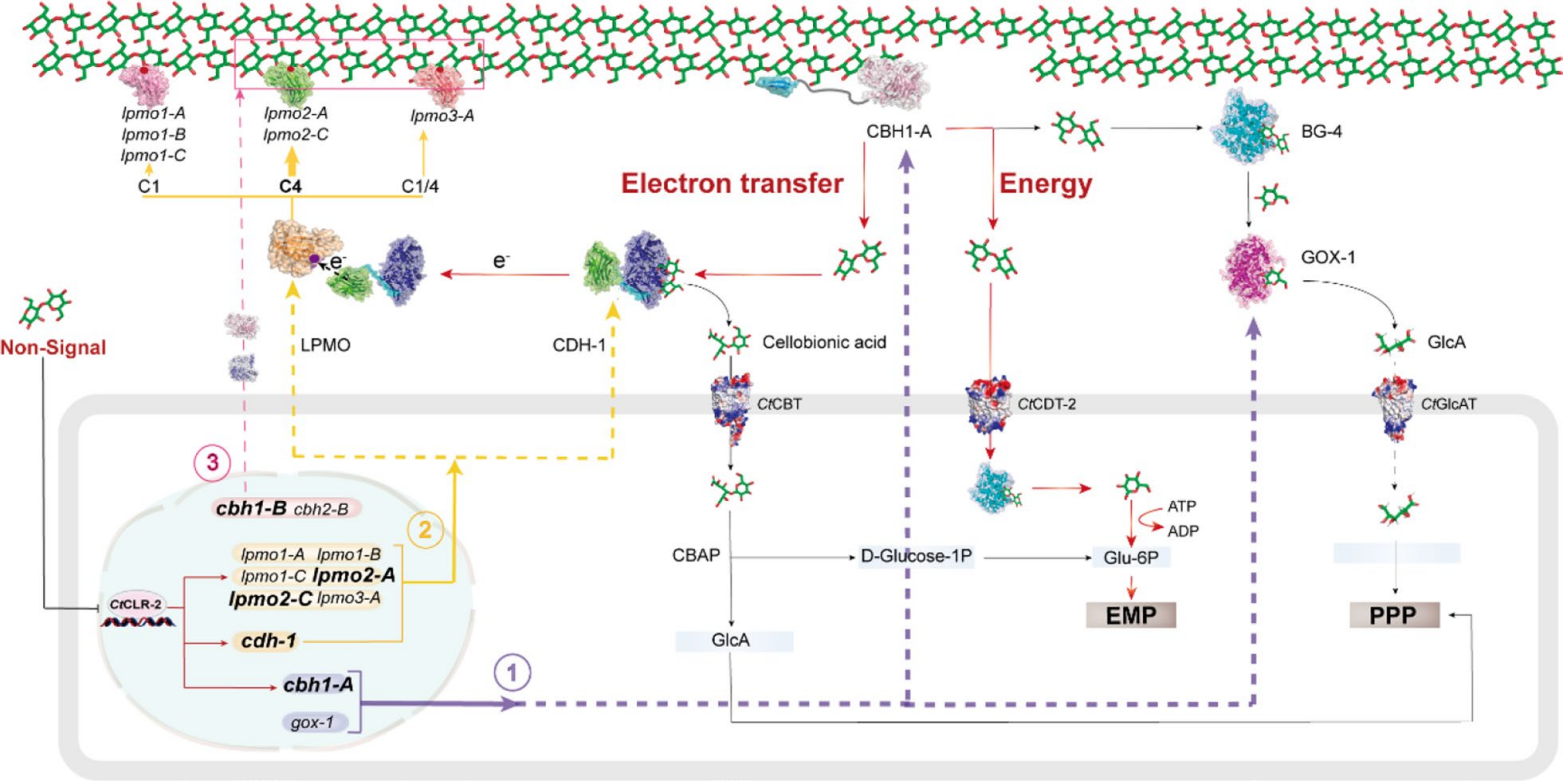

**Supplementary Information:**

The online version contains supplementary material available at 10.1186/s13068-026-02741-x.

## Background

Plant biomass with cellulose (a carbohydrate polymer composed of β-1,4-linked D-glucose units) as its main component is one of the most abundant renewable resources for sustainable bioenergy and materials production [1–4]. The highly crystalline structure of cellulose, maintained by intra- and intermolecular hydrogen bonding networks, forms a degradation-resistant barrier that limits enzyme accessibility and hinders its efficient conversion into value-added products [5, 6]. To address this, microbial systems have evolved synergistic enzyme cascades that disrupt crystallinity [7], but unlocking their full industrial potential requires deciphering the transcriptional and metabolic regulatory networks that govern enzyme expression and secretion.

Filamentous fungi have evolved sophisticated systems to degrade cellulose, utilizing a combination of hydrolases and oxidases [7–9]. Thermophilic fungi, in particular, offer unique advantages for biofuel production: their enzymes exhibit enhanced thermostability, broad pH tolerance, and resistance to chemical denaturants, traits that align with industrial fermentation conditions [10]. However, translating these properties into economically viable biofuel processes requires precise understanding of how fungi regulate enzyme systems—including optimal composition, stoichiometry, and secretion timing—to maximize synergistic efficiency [9]. Limitations in systematic understanding of these regulatory networks, however, hinder the rational optimization of industrial enzyme cocktails, slowing progress toward cost-effective lignocellulosic biofuels [11].

*Chaetomium thermophilum*, a thermotolerant fungus capable of degrading microcrystalline cellulose (MCC) with high efficiency, has emerged as a promising model for studying plant biomass conversion [12–16]. Its coding sequences share 90% similarity with *Thermothelomyces thermophila* and 80% with non-thermophilic species *Sordaria macrospora* and *Neurospora crassa* [15]. Genomic analysis confirms a complete cellulose-degrading arsenal: 5 cellobiohydrolases (CBHs), 10 endoglucanases (EGs), 8 β-glucosidases (BGs), 2 cellobiose dehydrogenases (CDHs; CAZy AA3_1), 8 glucose-methanol-choline (GMC; CAZy AA3_2) oxidoreductases, and 18 lytic polysaccharide monooxygenases (LPMOs) with specialized C1-, C4-, or dual-oxidation activity, a key for disrupting microcrystalline cellulose (MCC) [13, 17, 18]. LPMOs rely on electron donors, specifically CDHs and GMC oxidases, to drive oxidative cleavage: these systems dehydrogenate glucose and cellobiose to fuel LPMO activity, producing gluconic acid (GlcA) and cellobionic acid (CBA) as byproducts, while their synergy with hydrolases (for example, CBHs) directly dictates MCC degradation efficiency [19–25]. Notably, *C. thermophilum* achieves superior MCC degradation despite secreting lower total cellulase levels than industrial strains like *Trichoderma reesei*, implying a streamlined regulatory network that optimizes enzyme deployment, a trait highly desirable for reducing industrial enzyme costs [16].

Filamentous fungi regulate cellulases via conserved transcription factors, notably Clr-1, Clr-2 (including its homologues ClrB/ManR), and XlnR (Xlr1/Xyr1) [6, 26, 27]. In some fungi (e.g., *N. crassa*), Clr-1 senses cellobiose to activate Clr-2, thereby indirectly regulating cellulase genes, while Clr-2 acts directly on cellulase gene transcription [26, 28–30]. XlnR functions as a global regulator, governing the expression of both xylanases and cellulases in *Aspergillus niger* and *Aspergillus oryzae* [31, 32]. Notably, their regulatory roles are conserved yet species specific: Clr-2 is critical for cellulase production in *T. reesei* and *Thermothelomyces thermophilus*, whereas ClrB (a Clr-2 homologue) plays only a minor role in *Penicillium verruculosum* [33]. These transcription factors are triggered by polysaccharide degradation byproducts, and their inducer specificities differ significantly among species: D-xylose acts as the inducer for *A. niger*; gentiobiose, cellobiose, and sophorose serve as the specific inducers for *Penicillium* spp., *Aspergillus nidulans,* and *T. reesei*, respectively; and multiple inducers can activate the relevant pathway in *P. verruculosum* [33, 34]. Previous experiments revealed that 1% (w/v) cellobiose fails to induce cellulase expression in *C. thermophilum* [13]. This interplay between conservation and species specificity raises two key questions: Do thermophiles like *C. thermophilum* possess unique transcription factor synergistic mechanisms adapted to high-temperature cellulose degradation? And does its failure to induce cellulase expression in response to cellobiose stem from reducing sugar concentration inhibition or alternative activation pathways/signal molecules derived from MCC hydrolysis?

Here, building on our group’s previously established integrated omics approach for investigating cellulase expression and secretion across different carbon sources, we further integrate transcription factor analysis, gene-targeted knockout analysis via homologous recombination, and product metabolic network analysis to gain deeper insights into the regulatory and metabolic networks underlying efficient cellulose degradation in* C. thermophilum*. We focus on the folllowing: (1) Identifying key transcription factors governing the expression of cellulose-degrading genes and characterizing their regulatory modalities; (2) elucidating the temporal secretion patterns of these enzymes and their synergistic effects in disrupting cellulose crystallinity; and (3) the metabolism of oxidative products (CBA/GlcA) and their impact on pH regulation. By elucidating these mechanisms, this study deciphers *C. thermophilum*’s efficient cellulose degradation network, offering optimization principles for converting agricultural waste into biofuels and advancing sustainable biorefineries, while deepening insights into carbohydrate degradation in thermophilic fungi.

## Materials and methods

### Strains and culture conditions

*C. thermophilum* CGMCC3.17990 was used as the research strain. Spores were stored in 40% (v/v) glycerol at −80 °C. Minimal medium (MM; 1 L) comprising NaNO_3_ (5.95 g), KH_2_PO_4_ (1.497 g), KCl (0.522 g), MgSO_4_·7H_2_O (0.493 g), 1 mL of trace-element solution (76 mM ZnSO_4_, 178 mM H_3_BO_4_, 25 mM MgCl_2_, 18 mM FeSO_4_, 7.1 mM CoCl_2_, 6.4 mM CuSO_4_, 6.2 mM NaMoO_4_, 174 mM EDTA), and 1% (w/v) of different carbon sources (including cellobiose and MCC) was used to culture *C. thermophilum* at 50 °C with shaking at 200 rpm; the initial pH of the medium is 6.0.

### Mycelial biomass, MCC degradation efficiency, reducing sugars, protein concentration, and enzyme activity assays

Biomass and MCC degradation efficiency. Mycelia were separated from the supernatants (containing insoluble MCC) using a 100-mesh sieve, then rinsed thoroughly with distilled water. Supernatants were centrifuged at 12,000 × *g* for 15 min to pellet MCC. Both mycelia and MCC were dried at 60 °C to constant weight. Biomass and MCC degradation efficiency were calculated by gravimetric analysis. Culture supernatants were harvested every 12 h over a 5-day cultivation period to determine pH, concentrations of reducing sugars and proteins, as well as activities of CBH, CDH, and LPMO. For each biological replicate, all cultivation parameters were measured three times (with three technical replicates per measurement). pH was determined using a pH meter (Rex Electric Chemical, Shanghai, China).

Reducing sugars were measured through the 3,5-dinitrosalicylic acid (DNS) method [13]. The reaction mixture of filtered supernatant (1 mL) and DNS reagent (800 μL) was terminated by submerging the tube in boiling water for 10 min. Water was added to a total volume 10 mL, and absorbance at 540 nm was measured using a microplate reader (Puyuan Instruments, Shanghai, China). Standard curves were prepared using glucose over a concentration range of 0–10 mg/mL. One unit of enzyme activity (IU) is defined as the amount of enzyme required to release 1 µmol of reducing sugar per minute from the substrate.

Protein concentrations were determined using a Modified Bradford Protein Assay Kit (Sangon Biotech Co., Ltd., Shanghai, China). Supernatant (150 μL) and Bradford working solution (150 μL) were mixed at a 1:1 ratio, incubated at room temperature for 5 min, and absorbance at 595 nm was measured using a microplate reader. Bovine serum albumin (BSA) standard curves were prepared over a concentration range of 0–0.1 mg/mL.

The *p*-nitrophenyl-β-D-cellobioside (*p*NPC; Aladdin Biochemical Technology Co., Ltd, Shanghai, China) was used as the substrate for CBH activity measurements. Reaction mixtures (100 μL supernatant and 50 μL *p*NPC, 1 mg/mL) were incubated at 50 °C for 30 min, then quenched with 150 μL 1 M Na₂CO₃. Absorbance at 420 nm was measured using a microplate reader. Heat-inactivated enzyme served as a control. Gluconolactone (1 mg/mL; Sangon Biotech) was added to the* p*NPC solution to inhibit the activity of β-glucosidase. One unit (IU) of enzyme activity is defined as the amount of enzyme that generates 1 μmol of p-nitrophenol (*p*NP) from the hydrolysis of *p*NPC per minute.

CDH activity was measured using 2,6-dichloroindophenol (DCIP; Macklin Biochemical Co., Ltd., Shanghai, China). The reaction mixture consisted of 600 μL sodium hydrogen phosphate/citric acid buffer (pH 5.0), 50 μL 300 mM lactose, and 50 μL 3 mM DCIP, and was incubated at 30 °C for 30 min. After adding 100 μL of the culture supernatant, the absorbance at 520 nm was measured immediately, and a second measurement was taken after 3 min of incubation at 30 °C. Heat-inactivated enzyme served as a control. One IU of enzyme activity is defined as the amount of enzyme required to reduce 1 μmol of DCIP per minute.

LPMO activity was determined using 2,6-dimethylphenol (2,6-DMP; Sigma-Aldrich LLC, St. Louis, MO, USA) [35]. A reaction mixture containing 100 μL 1 M phosphates buffer (pH 6.0), 100 μL 10 mM 2,6-DMP, 20 μL 5 mM H_2_O_2_ (Sigma-Aldrich) solution, and 780 μL H_2_O was incubated in a cuvette at 30 °C for 15 min. Protein sample (100 μL) was then added, and absorbance at 469 nm was recorded immediately and after 300 s.

### Detection of isozyme zymograms by native-polyacrylamide gel electrophoresis (PAGE)

Changes in xylanase and endoglucanase isozymes secreted by *C. thermophilum* grown on cellobiose and MCC were analyzed by native-PAGE. Substrates were 2% (w/v) carboxymethylcellulose (CMC; Sangon Biotech) or 2% (w/v) xylan (Futaste, Shandong, China) dissolved in 0.02 mol/L sodium phosphate/citric acid buffer (pH 6.0). A 10% (w/v) separation gel was used, with 20 μL sample loaded per well. Electrophoresis was performed at 4 °C. Following electrophoresis, gels were incubated with respective substrates (CMC at 60 °C or xylan at 70 °C) for 30 min, washed with deionized water, stained with 0.5%(w/v) Congo Red in 10%(v/v) ethanol for 20 min, and finally destained in 1 M NaCl solution 2–3 times (20 min per wash). Gel images were acquired using a CanoScan 9000F Mark Il scanner (Canon Inc., Tokyo, Japan).

### Detection of extracellular proteins by SDS-PAGE

Extracellular proteins were analyzed using 12% (w/v) SDS-PAGE gels [36]. Protein samples (15 μL) were loaded into the wells and electrophoresed at 80 V until the tracking dye entered the separating gel, followed by 180 V until the dye reached the gel bottom. After electrophoresis, gels were stained with Coomassie Brilliant Blue R-250 (Sangon Biotech) for 40 min, then destained using a solution of ethanol:acetic acid:water (1:1:8, v/v/v). Images were acquired with a CanoScan 9000F Mark II scanner.

### Bioinformatics analysis of transcription factors and transporters

A literature review was conducted to identify key transcriptional factors and transport proteins involved in cellulose degradation in filamentous fungi. The amino acid sequences of these proteins were then compared with those in the *C. thermophilum* database (https://mycocosm.jgi.doe.gov/Chathe1/Chathe1.home.html). AlphaFold 3 was employed to model the structures of these proteins (https://golgi.sandbox.google.com/about) and their structural similarity to known transcriptional factors was assessed (https://seq2fun.dcmb.med.umich.edu/TM-score/). The JASPAR database (https://jaspar.elixir.no/search?q=&collection=CORE&tax_group=fungi) was employed to predict transcriptional factor binding sites on gene promoters, and these predictions were further validated via homologous recombination-mediated gene-targeted knockout and real-time quantitative PCR (RT-qPCR).

### Deletion of *Ctclr-2* genes

The 1.5–2.0 kb upstream and downstream sequences of *Ctclr*-*2* gene were individually cloned from *C. thermophilum* genomic DNA through PCR amplification. These sequences were cloned into the linearized pCE-115 plasmids along with Hygromycin B gene fragments, generating recombinant pCE-115- *Ctclr*-*2* plasmids. *Escherichia coli* DH5α cells were used for cloning and propagate of the recombinant vectors. The cells were cultured in medium supplemented with kanamycin (100 μg/mL). The resulting plasmids were digested with EcoR-I (Thermo Scientific, Massachusetts, USA) and linearized before being introduced into *C. thermophilum* through polyethylene glycol (PEG)-mediated protoplast transformation [12], generating Δ*Ctclr*-*2* knockout strains. Hygromycin B (250 μg/mL) was used as a marker to screen recombinants. Genomic DNA was extracted for PCR and sequenced to verify that *Ctclr*-*2* gene was deleted. In this way, Δ*Ctclr*-*2* knockout strains were obtained. Primers used for amplification and verification are listed in Table S1.

### Enzyme synergy assay

An initial mixture was prepared by combining 5 µL of CuCl₂ (50 mM), 5 µL of ascorbic acid (100 mM), and 410 µL of citrate–phosphate buffer (20 mM, pH 6.0). For simultaneous addition, 30 µL of LPMO and 50 µL of CBH were added concurrently to the mixture. The reaction was incubated for 12 h, after which reducing sugar release was quantified by the DNS method, measuring absorbance at 540 nm (OD_540_). For sequential addition, 50 µL of CBH was first added to the mixture and incubated for 6 h. Subsequently, 30 µL of LPMO was added, and incubation continued for an additional 6 h prior to OD_540_ measurement through the DNS assay. In the single enzyme control reactions, the volume of the omitted enzyme was substituted with 10 mM Tris–HCl buffer (pH 6.0) to maintain a constant final reaction volume of 500 µL. All enzymes used were derived from the *C. thermophilum* genome, heterologously expressed in *Pichia pastoris* GS115 to yield crude extracts, and subsequently purified to homogeneity via nickel affinity chromatography for synergistic assays.

### Analysis of the transcriptome

*C. thermophilum* strains were cultured in MM with 1% (w/v) cellobiose or MCC (3 biological replicates per group), and 50 mL samples were taken and centrifuged at 8000 × *g* for 15 min at 4 °C. The supernatant was discarded and mycelia were quickly frozen in liquid nitrogen and stored at −80 °C for RNA sequencing (RNA-seq) analysis [13]. The samples were sent to Majorbio (Shanghai, China) for processing. Processed samples were then aligned to the reference genome of *C. thermophilum* (GenBank assembly version: GCF_000221225.1; source: https://www.ncbi.nlm.nih.gov/genome/?term=Chaetomium+thermophilum) using Hisat (with default parameters) by the company; alignment rates across all samples ranged from 94.95 to 96.32%.

Subsequent data analysis was performed via the Majorbio cloud platform (https://cloud.majorbio.com/page/tools/). Gene expression levels were quantified using RSEM (v1.3.3) on the platform, with expression indices calculated as fragments per kilobase of transcript per million mapped reads (FPKM) and transcripts per million (TPM). For differential expression analysis, raw read counts were analyzed via DESeq2 on the Majorbio platform, with parameters: Benjamini–Hochberg correction, adjusted *P* < 0.05. Cluster analysis of differentially expressed genes was performed using hierarchical clustering (average linkage).

Expression analyses by real-time quantitative PCR (RT-qPCR).

Total mRNA was extracted using Trizol reagent (Sangon Biotech) and cDNA synthesis was performed using FastKing gDNA Dispelling RT SuperMix FastKing reagent (Tiangen, China). Primers for RT-qPCR analysis are listed in Table S2. RT-qPCR was performed using a qTOWER3G instrument (Jena, Germany) using SYBR Green Pro Taq HS (AG, China). The *gAPDH* gene was used for data normalization. 3 biological replicates were conducted, each with three technical replicates.

### HPLC analysis of GlcA

The predominant GlcA found in supernatant was determined by high performance liquid chromatography (HPLC) [37]. The culture supernatants were centrifuged at 10,000 × *g* for 10 min and mixed 1:1 with Solution B (methanol: acetonitrile, 7:6 v/v). Protein precipitation was performed at −20 °C overnight. Following centrifugation (10,000 × *g*, 10 min), supernatants were nitrogen evaporated to remove organic solvents. The supernatants were filtered through a 0.22 μm sterile microfilter and 50 μL of the filtrate was injected into the HPLC system (Shimadzu LC-20AT, Tokyo, Japan). GlcA in supernatants was separated by 20 mM KH_2_PO_4_ elution buffer at pH 2.4 and liquid B. The standard of GlcA (Aladdin) was used for comparison with samples. Separation was achieved using a TSKgel Amide-80 column (2.0 × 25 mm, 5 µm; Tosoh Corporation, Tokyo, Japan) equipped with a UV detector (210 nm). Gradient elution was performed at 0.8 mL/min and 25 °C as follows: 10–15% organic phase (0–5 min); 15–20% organic phase (5–10 min); 20–30% organic phase (10–15 min); 30–35% organic phase (15–20 min); 35–60% organic phase (20–30 min); 70% organic phase (30–35 min); and 70–10% organic phase (35–40 min).

### Extracellular proteome analysis by mass spectrometry

Protein analysis was carried out using liquid chromatography-tandem mass spectrometry (LC–MS/MS) as previously described [13]. For each time point, 3 biological replicates were established; 300 mL of cultured supernatant was collected from each replicate, and the supernatants of the 3 replicates were combined and thoroughly mixed prior to subsequent processing. The mixed supernatant was concentrated to 10 mL using a 3 kDa cut-off membrane (Millipore, Massachusetts, USA). The concentrated protein was then precipitated by adding acetone supplemented with 10% (w/v) trichloroacetic acid (TCA; Sigma-Aldrich) and 0.1% (w/v) dithiothreitol (DTT; Sigma-Aldrich), followed by drying to a powder. A 50 μg sample of the protein powder was mixed with 50 μL buffer solution (0.5 M Tris–HCl, 2.75 mM EDTA, 6 M guanidine-HCl) and incubated at 37 °C for 2 h. Next, 50 μL of 1 M iodoacetamide (Sigma-Aldrich) was added, and the mixture was incubated in the dark for 1 h, then transferred to a Microcon YM-10 centrifuge tube (MilliporeSigma, Burlington, MA, USA), and washed with 25 mM NH_4_HCO_3_ (Acros Organics, Brussels, Belgium). After adding 4% (w/v) trypsin (Sigma-Aldrich), digestion was performed overnight at 37 °C. The digested samples were desalted using a C18 ZipTip (Millipore) and dissolved in double-distilled water. LC–MS/MS analysis was performed using a premier nano LC system (Shimadzu, Kyoto, Japan) coupled with an LTQ-Orbitrap Velos Pro ETD mass spectrometer (Thermo Fisher Scientific, Waltham, MA, USA). The detailed procedure was based on a previous report [13].

Raw MS/MS data were processed for protein identification and quantification using MaxQuant software. The protein sequence database was derived from the UniProt proteome of *C. thermophilum* (Entry: UP000008066; retrieved from https://www.uniprot.org/proteomes?query=Chaetomium+thermophilum), which was constructed based on the reference genome assembly GCF_000221225.1. Peptide and protein identifications were filtered with a false discovery rate (FDR) ≤ 1%. For downstream data analysis, the ChiPlot online tool (https://www.chiplot.online/) was utilized for differential protein expression analysis, functional enrichment analysis, and heatmap generation.

### Omics data submission and availability

The mass spectrometry proteomics data were submitted to the ProteomeXchange Consortium (https://www.iprox.cn//page/SCV017.html?query=IPX0013993000) via the iProX partner repository [38, 39], with the dataset identifier IPX0013993000.

The transcriptomic data were submitted to the Sequence Read Archive (SRA) database (https://submit.ncbi.nlm.nih.gov) [40] via the National Center for Biotechnology Information (NCBI), with the BioProject accession numbers PRJNA1355141 and PRJNA1355244.

## Results

### Dynamic changes in extracellular reducing sugars, protein levels, and activities of CBHs, CDHs, and LPMOs

**Fig. 1:**
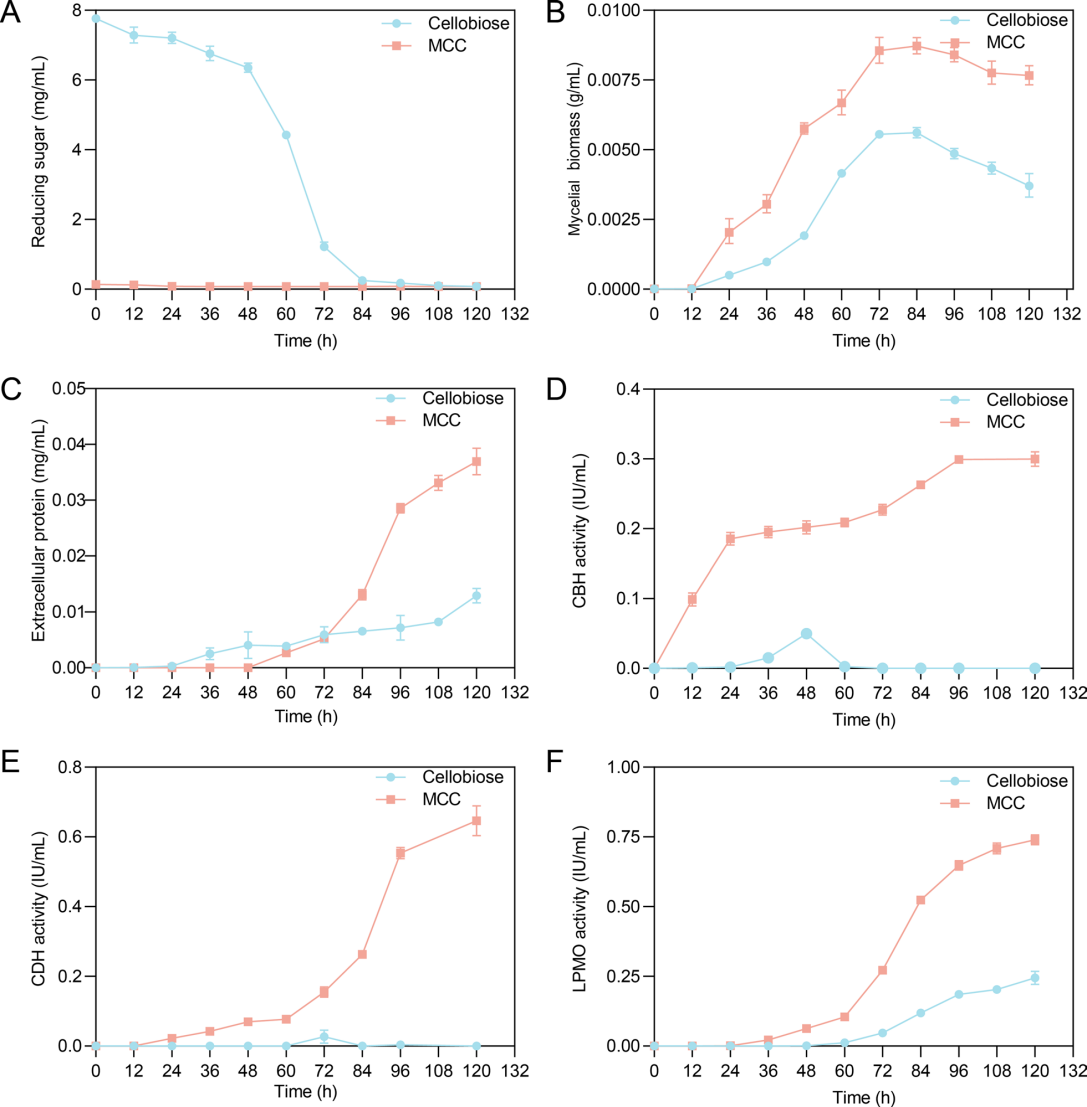
Dynamic changes in extracellular reducing sugar, biomass, protein, and enzyme activities of *C. thermophilum* cultured on cellobiose and MCC over a 5-day period. **A** Reducing sugar concentration (mg/mL) over time. **B** Mycelial biomass (g/mL) over time. **C** Extracellular protein content (mg/mL) over time. **D** CBH activity (IU/mL) over time. **E** CDHs activity (IU/mL) over time. **F** LPMOs Activity over time. All measurements are based on three biological replicates, with standard deviations indicated by error bars

Carbon source utilization patterns were characterized by monitoring extracellular reducing sugars, biomass, and enzyme activities. Extracellular reducing sugars in cellobiose cultures decreased by 15% within 48 h and became undetectable after 84 h, whereas MCC cultures showed no significant accumulation (Fig. 1A). *C. thermophilum* grown on MCC reached 9 mg/mL biomass—double that of cellobiose cultures (Fig. 1B). Biomass peaked at 72 h and then declined by 33.9% (cellobiose cultures) and 12.8% (MCC cultures) at 120 h relative to 72 h.

Extracellular protein concentration assays revealed no significant difference in protein levels between the two substrates before 72 h (Fig. 1C). However, after 72 h, extracellular protein levels in MCC cultures peaked at 0.37 mg/mL—fourfold higher than those in cellobiose cultures. The dynamic changes in extracellular protein content were consistent with the extracellular proteomic profiles (Fig. S1A). Proteomic profiling identified seven proteins commonly induced under both MCC and cellobiose conditions, among which proteins 2 and 4 were significantly upregulated by MCC. Notably, MCC specifically induced four extracellular proteins, with protein 3 being the most abundantly secreted. In contrast, cellobiose specifically induced only one extracellular protein (Fig. S1A).

LPMO activity in MCC-induced cultures was 3.1-fold higher than in cellobiose cultures at 120 h, with CDH and CBH activities peaking at 0.65 IU/mL and 0.31 IU/mL, respectively. However, CDH and CBH activities were negligible in cellobiose cultures at 120 h. CBHs were secreted early (< 12 h), with activity peaking at 96 h following a secondary increase after 60 h (Fig. 1D). CDHs and LPMOs were secreted later, with significant expression after 72 h of MCC cultivation (Fig. 1E, F). These results align with extracellular protein profiles (Fig. S1B).

### Identification of transcription factor homologs regulating cellulases

**Fig. 2:**
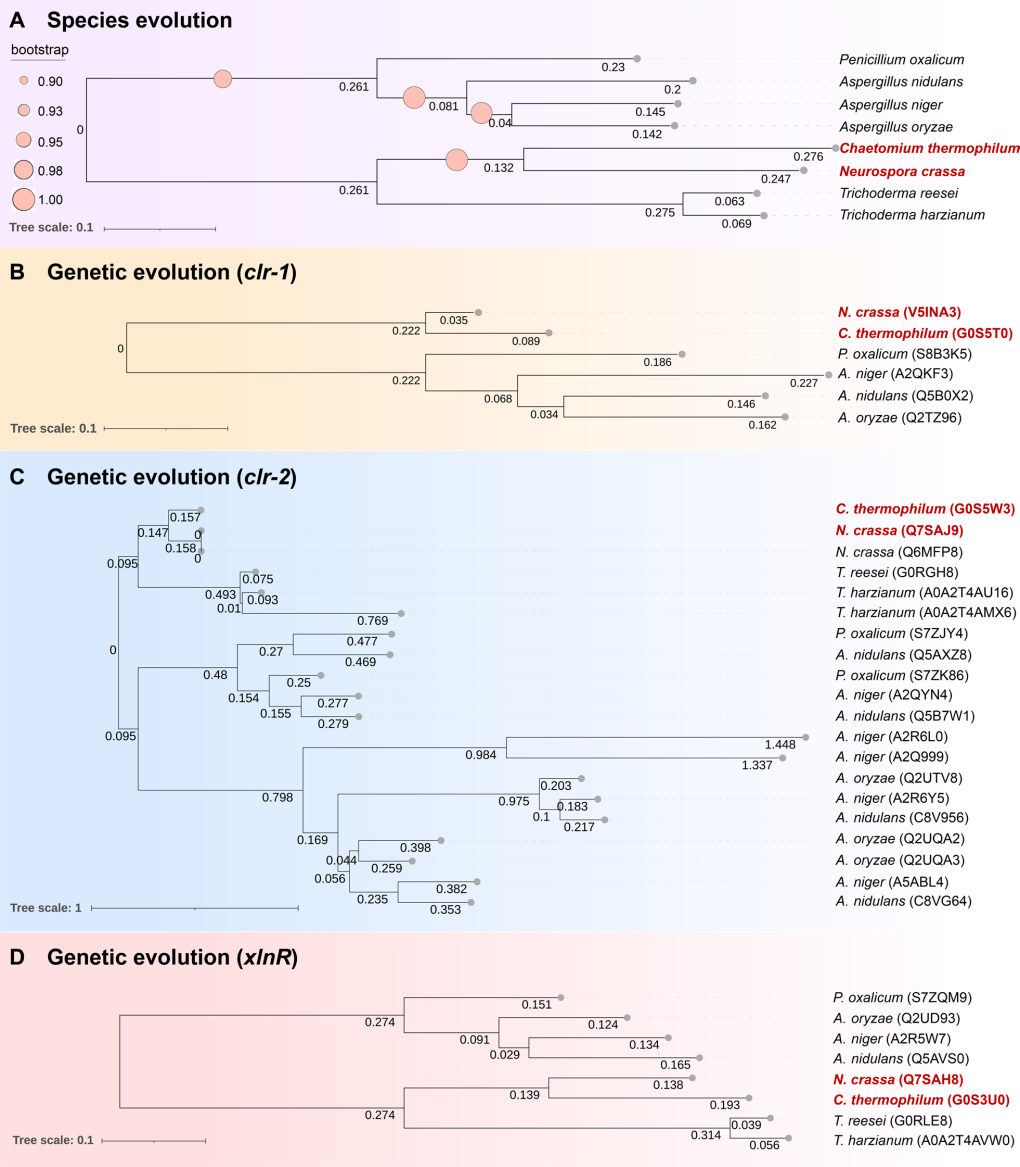
Comparative phylogenetics of species and cellulolytic regulators. **A** Species phylogenetic tree with bootstrap values (0.90–1.00) denoting branch reliability. **B** Phylogenetic tree of the CLR-1 gene family. **C** Phylogenetic tree of the CLR-2 gene family. **D** Phylogenetic tree of the XlnR gene family

The transcription factors Clr-1 and Clr-2 from *T. thermophilus* or *N. crassa*, as well as XlnR from *Talaromyces versatilis*, regulate cellulase gene transcription [29, 41, 42]. To identify key transcription factors governing cellulase expression in *C. thermophilum* and resolve their evolutionary relationships, we performed sequence homology searches combined with phylogenetic analyses. Phylogenetic analysis at the species level showed *C. thermophilum* clusters closely with *N. crassa* with high bootstrap support (≥ 0.90) (Fig. 2A).

Sequence homology searches revealed proteins G0S5T0, G0S5W3, and G0S3U0 shared > 83% sequence coverage and > 74% similarity with *N. crassa* Clr-1 (V5INA3), Clr-2 (Q7SAJ9), and XlnR (Q7SAH8), respectively (Table 1). Gene family-level phylogenetic analysis confirmed these as orthologs: G0S5T0 to *N. crassa* Clr-1, G0S5W3 to Clr-2, and G0S3U0 to XlnR (Fig. 2B–D). No paralogs of *clr-1*, *clr-2*, or *xlnR* were found in *C. thermophilum*. These proteins were designated *Ct*Clr-1, *Ct*Clr-2, and *Ct*XlnR in *C. thermophilum*, respectively.

**Table 1:** Prediction and sequence analysis of transcription factors involved in cellulose degradation

Gene name	UniProt ID	Organism	Sequence			Zn_2_Cys_6_ sequence	DNA binding sequence
			Identity (%)	Cover (%)	Total score		
*Ctclr-2*	CTHT_0033840	*C. thermophilum*	81.6	83	2976	ACAECKRRKIRCDGQQPCGQCLSSRAPKRCFY	CGGN_11_CCG
*clr-2*	NCU08042	*N. crassa*				ACAECKRRKIRCDGQQPCGQCLSSRAPKRCFY	
*Ctclr-1*	CTHT_0033500	*C. thermophilum*	84.2	92.7	2768	N/A	CGGN5CGGNCGG
*clr-1*	NCU07705	*N. crassa*				ACEVCRSRKSRCDGTKPKCKLCTELGAECIY	
*Ctclr-3*	CTHT_0020530	*C. thermophilum*	64.6	66.7	2315	N/A	Unknown
*clr-3*	NCU05846	*N. crassa*				N/A	
*Ctclr-5*	CTHT_0029070	*C. thermophilum*	80.4	73.6	3001	N/A	Unknown
*clr-5*	MYCTH_2301131	*T. thermophilus*				N/A	
*CtxlnR*	G0S3U0	*C. thermophilum*	74.6	87.2	3387	ACDQCNQLRTKCDGQQPCAHCIEFQLTCEY	GGCTARA/GGCWWWW/GGNTAAA
*xlnR*	3878781	*N. crassa*				ACDQCNQLRTKCDGQHPCAHCIEFGLGCEY	

Sequence and structural analysis showed *Ct*Clr-1 lacks the Zn_2_Cys_6_ domain (Table 1). The Zn_2_Cys_6_ domain of *Ct*XlnR differs from that of *N. crassa* XlnR, with His110 and Gly119 substituted by glutamine residues. Conversely, the Zn_2_Cys_6_ domain of *Ct*Clr-2 shares 100% similarity with Clr-2. AlphaFold 3 modeling of *Ct*Clr-2 interactions with the *cbh1-A* promoter region demonstrated a binding mode consistent with the previously reported GAL4-type Clr-2, which forms dimers to recognize CG-rich DNA motifs (Fig. S2) [43]. *CtClr-2* binding to the CBH1-A promoter site was consistent with the predicted sequences listed in Table S3.

### Regulatory role of core transcription factor *Ct*Clr-2 in cellulase degradation

**Fig. 3:**
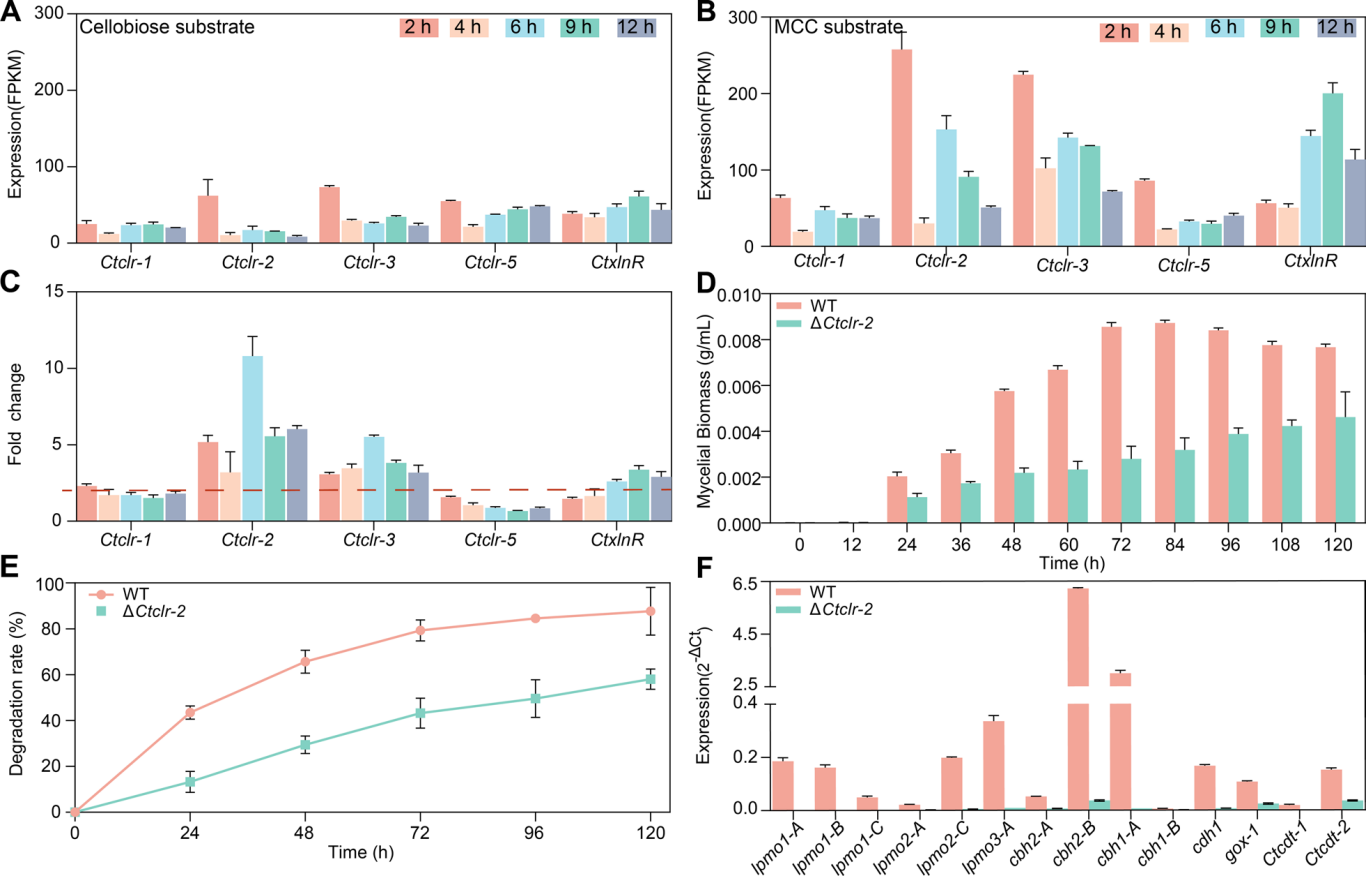
Transcriptional and phenotypic characterization of WT and Δ*Ctclr*-*2* strains related to cellulose degradation. **A** Transcription levels (FPKM) of transcriptional factors induced by cellobiose at 2, 4, 6, 9, and 12 h. **B** Transcription levels (FPKM) of transcriptional factors induced by MCC. **C** Fold change in transcription of transcriptional factors between MCC and cellobiose conditions. The dashed red line denotes a fold change of 2 (no differential expression). **D** Mycelial biomass (g/mL) comparison between WT and Δ*Ctclr*-*2* strains over time. **E** MCC degradation rates (%) of WT and Δ*Ctclr*-*2 *strains. **F** Transcription levels of cellulose degradation-related oxidase and hydrolase genes (detected by RT-qPCR, calculated via the 2^−ΔCt^ method). All data are presented as means ± standard deviations from three biological replicates

Further analysis was performed on the temporal dynamics of expression levels of different transcription factors. RT-qPCR analysis showed that MCC substrates significantly upregulated the transcription of *CtxlnR*, *Ctclr-1*, and *Ctclr-2* compared to cellobiose (Fig. 3A, B). *CtxlnR* was strongly induced by MCC at 6 h, with transcription levels 3.5-fold higher than in cellobiose cultures. *Ctclr-1* and *Ctclr-2* exhibited high transcription levels within 2 h, and *Ctclr-2* transcription at 6 h was 10.8-fold higher in MCC cultures than in cellobiose cultures (Fig. 3C). No canonical *Ct*Clr-1 binding motif (CGGN_5_CGGNCCG) was detected in the *Ctclr-2* promoter (Table S3).

To clarify *Ct*Clr-2 function, Δ*Ctclr-2* knockout strains were constructed (Fig. S3A). No significant differences in mycelial morphology were observed between the WT and Δ*Ctclr-2* strains (Fig. S3B), but Δ*Ctclr-2* grew slower on MCC than the WT (Fig. S3C). Notably, mycelial autolysis of the WT initiated at 72 h post-inoculation and increased markedly by 96 h, whereas no obvious autolysis was detected in Δ*Ctclr-2* during the same period. At 120 h, Δ*Ctclr-2* showed a 50% reduction in maximum biomass and a 30% decrease in MCC degradation efficiency relative to the WT (Fig. 3D, E).

To clarify the transcriptional regulatory role of *Ct*Clr-2, we used RT-qPCR to quantify transcript levels of cellulase genes in both WT and Δ*Ctclr-2* strains culture on MCC. The results showed that the transcription of all cellulase genes was significantly downregulated except for *cbh1-B* and *cbh2-B*. Specifically, *lpmo1-A*/*B*/*C*, *lpmo2-A*/*C*, *lpmp3-A*, *cdh-1,* and *cbh1-A* showed no detectable transcription (Fig. 3F), and their promoter regions contained *Ct*Clr-2 binding motifs (CGGN₁₁CCG; Table S3) [44]. Notably, *cbh2-A*, *gox-1* (glucose oxidase, a member of the GMC oxidase family), *Ctcdt-1*, and *Ctcdt-2* showed reduced expression despite lacking binding sites. In addition to the above transcription factors, no Clr-4 homolog was identified in *C. thermophilum*. *Ctclr-3* (G0S3C6) exhibited 3–fivefold increased expression under MCC compared to cellobiose, whereas *Ctclr-5* (G0S819) remained relatively stable (Fig. 3C).

### Transcriptomic expression profiling under different carbon sources

**Fig. 4:**
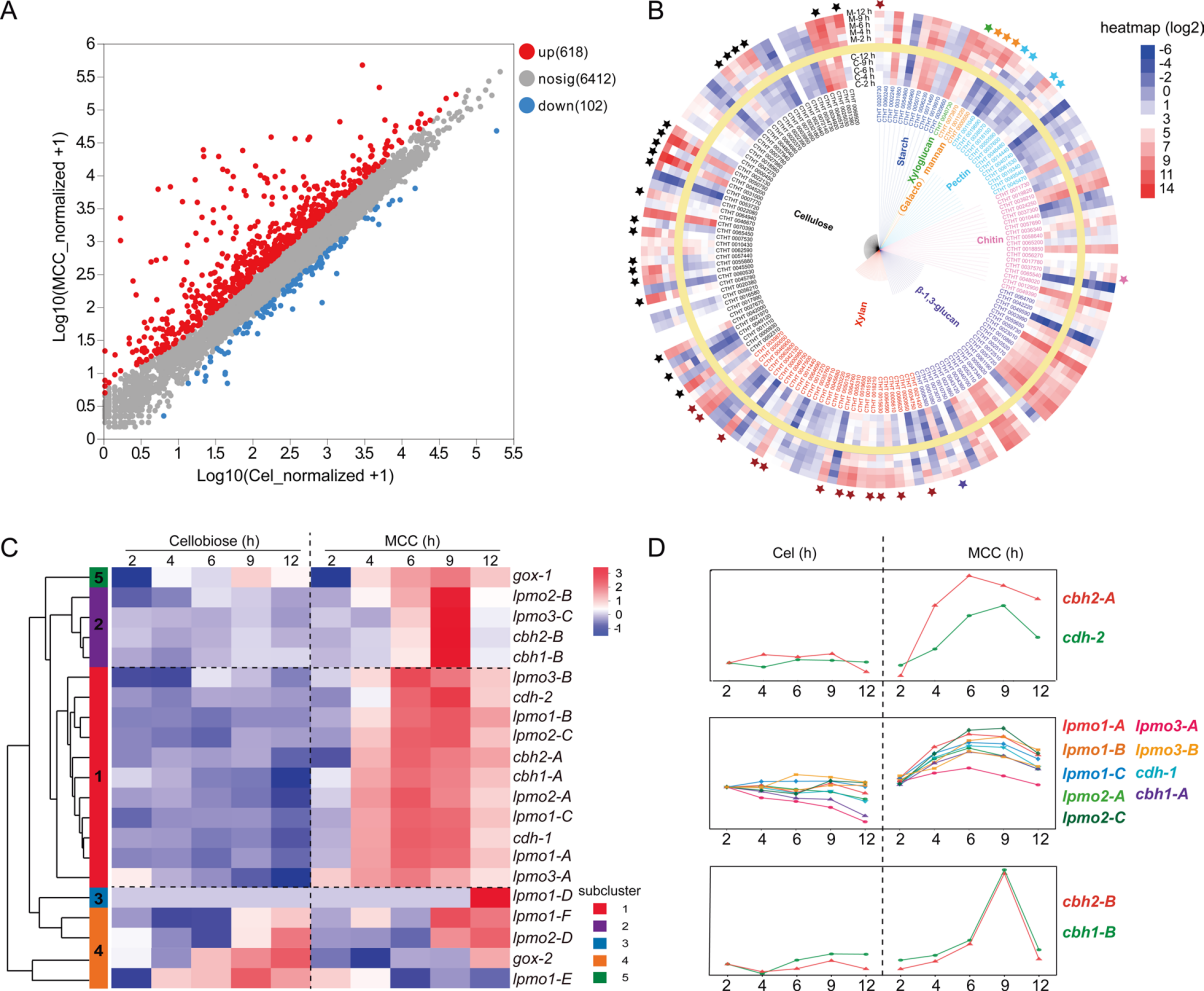
Transcriptomic profiling of carbon source-dependent gene regulation. **A** Scatter plot of differential gene expression between MCC and cellobiose. **B** Circular heatmap illustrating transcriptional variation of carbohydrate degradation-related genes across MCC and cellobiose over time. A yellow ring separates cellobiose (inner) and MCC (outer), with incubation time increasing radially. Asterisked genes show significantly higher transcription in MCC. The color scale represents relative transcript abundance. **C** Normalized hierarchical clustering of cellulose degradation-associated hydrolase and oxidase genes. The left color bar denotes enzyme subfamilies, and the right scale reflects relative transcript abundance. **D** Temporal transcription pattern analysis via the sparse topology and energy management (STEM) algorithm. Only co-transcribed gene profiles (among 50 tested patterns) are shown; single-gene or non-significant trends are excluded

RNA-seq of the WT strain grown on MCC versus cellobiose detected 7,474 transcribed genes, with 720 differentially expressed (618 upregulated; Fig. 4A). Among these upregulated differentially expressed genes, 41.2% of cellulases, 42.9% of xylanases, 30.8% of pectinases, and 33.3% of mannanases were significantly induced in MCC cultures (Fig. 4B).

Transcriptional pattern analysis divided cellulose degradation-related genes into four clusters (Fig. 4C). *Ct*Clr-2 targets (*cbh1-A*, *lpmos*, *cdh-1*) co-clustered in Cluster 1 with peak expression at 6 h under MCC. The *cbh1*/*2-B* genes showed delayed induction compared with *cbh 1*/*2-A*. Overall transcription of CBHs was ~ twofold higher than LPMOs. The *gox-1* formed a distinct Cluster 5, expressed under both carbon sources but preferentially induced by MCC (peak at 9 h). These findings were further validated by RT-qPCR (Fig. S4).

To further analyze the consistency of temporal trends in cellulose degradation-related hydrolases and oxidases, temporal trend analysis was applied to these enzymes (Fig. 4D). Consistent with the transcriptional regulatory analysis, *lpmo1-A/B/C*, *lpmo2-A/C*, *lpmo3-A*, *cbh1-A*, and *cdh-1* (CtClr-2 targets) were classified into profile 2. Unlike the hierarchical clustering results, the *cbh2-A* gene was absent from profile 2. Meanwhile, *cbh1-B* and *cbh2-B* genes, not regulated by *Ct*Clr-2, were separately classified into profile 3.

### Temporal proteome profiling of cellulolytic enzymes

**Fig. 5:**
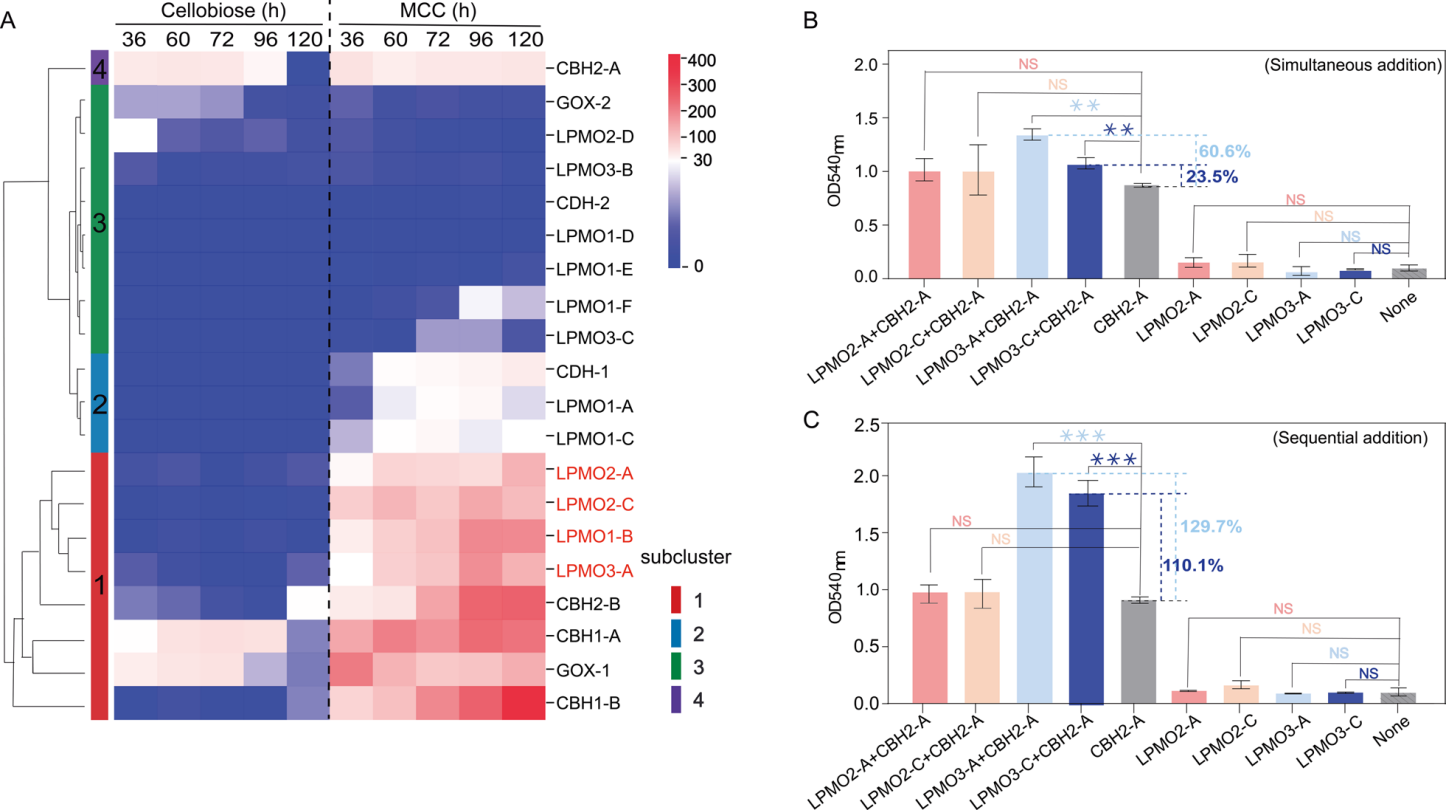
Analysis of proteomes with different carbon sources and enzymatic synergy via differential enzyme addition. **A** Non-normalized hierarchical clustering heatmap of secreted cellulose-degrading hydrolases and oxidases in *C. thermophilum* cultured on cellobiose and MCC at 36, 60, 72, 96, and 120 h. The left color bar indicates enzyme subfamilies, and the right scale reflects relative protein abundance. **B** Impact of simultaneous addition of oxidase LPMOs and hydrolase CBH2-A on reducing sugar production (OD₅₄₀) from CMC degradation, with controls as “only CBH2-A” and “None.” Percentage values indicate relative changes versus controls. **C** Impact of sequential addition of LPMOs and CBH2-A on reducing sugar production from CMC degradation. Data are means ± SD (*n* = 3); *P* < 0.05 denotes statistical significance

Proteomic analysis revealed differential secretion of cellulose-degrading enzymes (Fig. 5A). Among 18 identified AA9 LPMOs, LPMO1-B (C1-oxidizing), LPMO2-A/C (C4-oxidizing), and LPMO3-A (C1/C4-oxidizing) were significantly secreted, with LPMO2-A/C exhibiting 1.6–1.8-fold higher abundance than other subfamilies (Table S4). CBH1 family members were secreted at markedly higher levels than CBH2 (Fig. 5A). Additionally, in vitro synergy assays of LPMOs and hydrolases from *C. thermophilum* showed that LPMO2 addition did not increase reducing sugar release beyond CBH2-A alone; LPMO3-A and LPMO3-C enhanced yields by 60.6% and 23.5%, respectively (Fig. 5B).

Electron donor systems exhibited distinct secretion dynamics: GOX-1 was constitutively expressed, while CDH-1 was specifically induced by MCC (Fig. 5A). Proteomics identified a three-stage secretion cascade: CBH1-A was secreted first, followed by oxidoreductases (LPMOs, CDH-1; ~ 60 h delay), and finally CBH1/2-B (peaking at 120 h; Fig. 5A). This aligned with the secondary increase in extracellular CBHs activity (Fig. 1D). In vitro validation showed sequential addition of LPMOs and CBH2-A enhanced synergy compared with simultaneous addition, with increase of 69.1% for LPMO3-A and 86.6% for LPMO3-C (Fig. 5B, C).

Integrated omics analysis revealed that *C. thermophilum* encodes 189 carbohydrate-degrading genes (including 51 cellulase-encoding genes), of which 114 (32 from the cellulase subset) harbor signal peptides. Upon substrate induction, transcriptomics showed that 98 of these 114 signal peptide-containing genes (27 cellulase-encoding ones) were transcriptionally induced. However, the proteome detected only 90 corresponding extracellular proteins (25 cellulases; Supplementary Table S5).

### Inducers and metabolic network of cellulose degradation

**Fig. 6:**
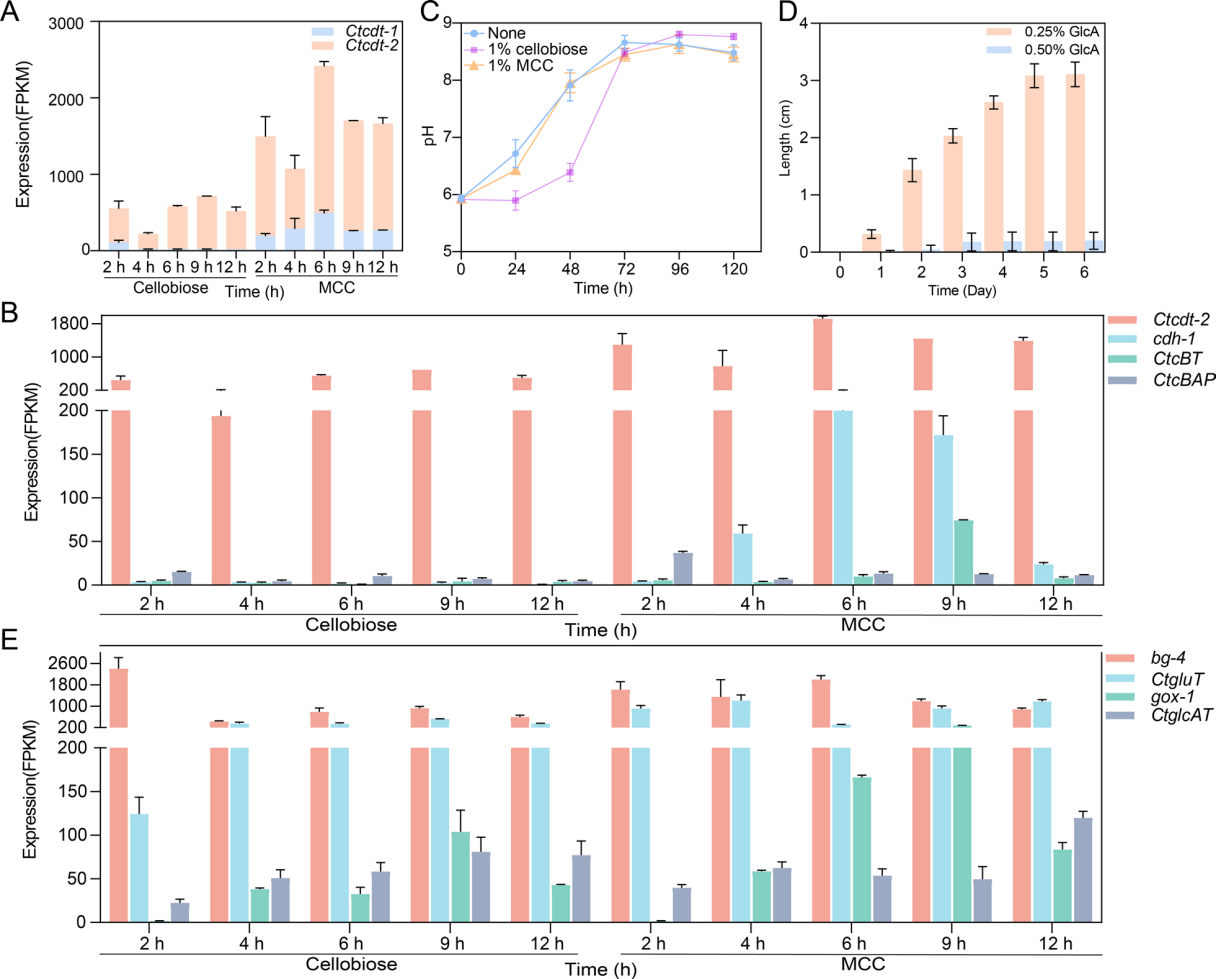
Transcriptional and physiological dynamics of *C. thermophilum* related to sugar acid metabolism across carbon sources. **A** Transcription levels (FPKM) of *Ctcdt-1* and *Ctcdt-2* under cellobiose and MCC at 2, 4, 6, 9, and 12 h. **B** Transcription levels (FPKM) of genes associated with cellobionic acid (CBA) transport and metabolism. **C** Variations in culture medium pH. **D** The influence of GlcA concentration on the growth of *C. thermophilum*. **E** The transcriptional levels of genes related to the production and transportation of glucose or GlcA. All data are presented as means ± SD from 3 biological replicates (*n* = 3)

To verify whether cellulase expression in *C. thermophilum* is activated by the CDT-mediated cellobiose internalization mechanism common in fungi, the expression of transporter gene *Ctcdt* and cellulase genes was determined. Transcriptomic analysis revealed MCC-induced expression of *Ctcdt-1* (CTHT_055680) and *Ctcdt-2* (CTHT_0057310), with *Ctcdt-1* exhibiting MCC-specific responsiveness in *C. thermophilum* (Fig. 6A). However, cellobiose induction experiments (concentration gradient: 0.01–1% (w/v)) demonstrated that cellobiose failed to efficiently induce the transcription of cellulose degradation-related genes in *C. thermophilum*, irrespective of concentration (Fig. S5A). Even at a low concentration of 0.01% (0.1 mg/mL), cellobiose failed to induce these target genes effectively—this contrasts sharply with MCC, which achieved robust induction despite a comparable reducing sugar concentration (0.14 mg/mL) (Fig. S5A).

*C. thermophilum* cellulose degradation yields oligosaccharides and sugar acids through oxidative and hydrolytic processes. To decipher the intracellular metabolism of sugar acids (predominantly cellobionic acid, CBA; and GlcA) in *C. thermophilum*, sequence analysis identified a CBA transporter gene (*CtcBT*, CTHT_0047580) and a phosphorylation-related gene (*CtcBAP*, CTHT_0019620) (Table S6), Both exhibited MCC-specific induction congruent with *cdh-1* transcription (Fig. 6B), peaking at 9 h and 2 h, respectively. Medium pH dynamics showed significant acidification in cellobiose cultures, reaching pH 6.2 by 48 h (1.6 units below MCC and no-carbon groups; Fig. 6C), followed by recovery to pH 8.4 at 72 h. This coincided with 4.6-fold higher extracellular GlcA accumulation at 24 h (Fig. S6A) and early secretion of BG-4 and GOX-1 (but not CDHs; Fig. S5B).

Results regarding GlcA utilization and metabolism showed that *C. thermophilum’*s capacity for GlcA utilization as a sole carbon source resulted in maximum mycelial growth (3.1 cm diameter) at 0.25% (w/v) GlcA after 5 days, whereas 0.5% GlcA caused complete inhibition (Fig. 6D). Transcriptomic analysis demonstrated earlier induction of *bg-4*, *hgt* (high-affinity glucose transporter), and *CtglcAT* (a putative GlcA transporter; unpublished data) compared to *gox-1* (Fig. 6E). When the GlcA concentration was below 0.25%, *CtglcAT* transcription correlated positively with GlcA concentration (Fig. S6B).

## Discussion

Plant biomass degradation by fungi involves specialized strategies, with thermophiles like *C. thermophilum* offering unique insights into efficient lignocellulose breakdown under high temperatures.

### Carbon source utilization and adaptive secretion of cellulolytic enzymes

Soluble cellobiose was rapidly utilized within 48 h, while MCC was hydrolyzed gradually to sustainably release reducing sugars for biomass accumulation (double that of cellobiose cultures). Notably, accelerated mycelial growth at higher temperatures forms tightly twisted structures, exacerbating nutrient scarcity and hypoxia [45, 46]. These environmental stresses presumably trigger mycelial autolysis starting at 72 h in the late culture phase, reducing biomass, releasing intracellular proteins, and driving a sharp surge in extracellular protein content. However, compared to cellobiose, MCC’s recalcitrance supports a sustained nutrient supply for mycelial growth, delaying autolysis induced by such environmental changes. Knockout of *Ctclr-2*, the key cellulose-degrading gene, reduced MCC degradation and mycelial growth by over 30%. This further reduces the fungus’s nutrient and oxygen demands, delaying autolysis compared to the WT strain.

CBHs were secreted early (< 12 h), with activity peaking at 96 h following a secondary increase, whereas CDHs and LPMOs were expressed later. This sequential secretion aligns with in vitro synergy assays showing that sequential addition of LPMOs and CBH2-A enhanced reducing sugar release by 69.1–86.6% compared with simultaneous addition, mirroring observations in lignocellulose degradation by other fungi [47, 48] and reinforcing the evolutionary advantage of staggered enzyme secretion for optimizing substrate breakdown.

### Transcriptional regulation of cellulases: the central role of *Ct*Clr-2

Despite significant variations in target gene sets among filamentous fungi, transcription factor sequences and structures remain relatively conserved [30]. Homologs of *N. crassa* Clr-1, Clr-2, and XlnR were identified in *C. thermophilum*, but structural divergence was evident: *Ct*Clr-1 lacks the Zn_2_Cys_6_ domain, and *Ct*XlnR contains substitutions in its Zn_2_Cys_6_ domain, whereas *Ct*Clr-2 retains 100% similarity in this domain to *N. crassa* Clr-2. This conservation, coupled with AlphaFold 3 modeling showing *Ct*Clr-2 dimerization and binding to CG-rich motifs in the *cbh1-A* promoter [43], underscores *Ct*Clr-2’s central role. This conclusion is validated by Δ*Ctclr-2* knockout strains, which exhibited over 30% reduction in mycelial biomass and MCC degradation, and near-complete loss of transcription of key enzymes [44]. While the apparent absence of the Zn_2_Cys_6_ domain in *Ct*Clr-1 could reflect a bioinformatic artifact (e.g., incorrect gene modeling or genome sequencing gaps), lack of a canonical *Ct*Clr-1 binding motif in the *Ctclr-2* promoter strongly indicates *Ct*Clr-1 may not mediate indirect cellulase activation, unlike its homologs in *N. crassa* and *T. thermophilus* [41]. *Ct*XlnR, induced by MCC at 6 h (a time point when *Ct*Clr-2 is already regulating cellulase expression), shows functional divergence from XlnR in *T. reesei* (which regulates both cellulases and xylanases) and aligns more closely with *N. crassa* XlnR, lacking core cellulase regulatory activity [29, 49].

### Coordinated regulation of cellulolytic machinery

Omics analyses explain the coordinated regulation of cellulolytic machinery in *C. thermophilum*. *C. thermophilum* harbors 11,611 genes [15]. Among the 7,474 transcribed genes, 618 were upregulated by MCC, approximately 41.2% cellulases and 42.9% xylanases—reflecting coordinated induction of cellulose and hemicellulose degradation systems, a pattern conserved in fungi like *T. aurantiacus* and *Ganoderma applanatum* [50, 51]. In hierarchical clustering, *Ct*Clr-2 targets (*cbh1-A*, *lpmos*, *cdh-1*) clustered together, while *cbh1-B* and *cbh2-B* (not regulated by *Ct*Clr-2) formed distinct clusters, indicating subfunctionalization of cellulase isoforms. Furthermore, functional specialization among the 18 LPMOs was evident via differential secretion across substrates. Notably, proteomic data and in vitro synergy assays confirm a synergistic relationship between LPMO2/3 and CBH1. This synergistic relationship aligns with regioselectivity trends in fungi, where C4-oxidizing LPMO2 promote CBH1 activity and dual-functional LPMO3 universally boost efficiency [23, 52, 53]. The coupling of CDH-1 with LPMOs, facilitated by *Ct*Clr-2 co-regulation, highlights its role as a critical electron donor, consistent with genomic evidence linking *cdh* genes to expanded LPMO families [19, 20]. CDH-1 leverages its rapid electron transfer rate to activate LPMOs despite low secretion levels [54].

Compared with Li et al.’s previous work [13] and by integrating molecular biology techniques, we further revealed that *Ct*Clr-2 mediates the co-transcription of oxidases and hydrolases in *C. thermophilum*. Critically, this co-transcription does not drive synchronous secretion—reflecting a fungal-specific non-linear relationship between gene expression and protein secretion driven by post-transcriptional regulation [55–57]. This regulatory layer enables the staggered secretion of oxidases before hydrolases (as in *Laetisaria arvalis* [58]), which not only provides additional binding sites for subsequent hydrolases but also optimizes enzymatic synergy. During MCC degradation, initial CBH1-A secretion fulfills growth demands while generating reducing power for CDH-1/LPMOs activity; later, LPMO/CDH-1 release (translationally sustained by persistent MCC) produces oxidation intermediates that trigger CBH1-B/CBH2-B expression, thereby converting glycans into metabolizable oligosaccharides [59]. This temporal coordination, rooted in transcriptional co-regulation of *cdh-1*, *lpmos*, and *cbh1-A*, explains why sequential enzyme addition outperforms simultaneous mixing for enhancing degradation efficiency, highlighting it as an evolutionary strategy for filamentous fungi to maximize complex substrate breakdown [60]. Compared with previous studies (transcription factors or enzymes) [7, 32, 33, 55, 61, 62], systematic analysis of the global network underlying cellulose degradation in *C. thermophilum* can more accurately and efficiently guide fungal metabolic engineering, thereby improving the efficiency of plant biomass conversion in relevant industrial applications.

### Induction mechanisms and metabolic adaptation to cellulose byproducts

Cellobiose induces cellulases via CDT-mediated internalization in *N. crassa* [63]; yet even at low concentrations (0.1 mg/mL), it failed to induce cellulase genes in *C. thermophilum*. By contrast, MCC triggers robust cellulase induction with comparable reducing sugar levels, ruling out high reducing sugar concentrations as the cause of failed cellulase activation. Instead, we speculate that MCC-derived oligosaccharides (e.g., cellotriose, cellopentaose) may act as signaling molecules to initiate cellulase gene transcription, a mechanism observed in *Phanerochaete chrysosporium* [64, 65]. Fungal sugar transporters exhibit significant complexity [66]. In *A. nidulans*, CltA transports cellobiose and CltB is mainly responsible for substrate sensing and signaling [67]. *C. thermophilum* harbors two homologous transporters, *Ct*CDT-1 and *Ct*CDT-2. Unlike CDT-2 in *M. thermophila*, which acts as a xylodextrin transporter [68], *Ct*CDT-2 expression correlates strongly with cellobiose, indicating its primary role as a cellobiose transporter. Unlike *N. crassa*’s CDT-2 and *A. nidulans*’ CltB, *Ct*CDT-2 does not activate cellulase genes; it mainly transports cellobiose and sustains fungal growth [64]; *Ct*CDT-1 is specifically induced by MCC, implying that it may be primarily responsible for substrate sensing and signaling.

Metabolic adaptation to cellulose degradation byproducts is another hallmark. *C. thermophilum* metabolizes cellobionic acid through *Ct*CBT and *Ct*CBAP, both specifically induced by MCC—consistent with CDH’s specific induction. GlcA is metabolized via the pentose phosphate pathway [69, 70]; however, concentrations ≥ 0.5% inhibit growth, likely adaptive fine-tuning to avoid toxicity. Temporal links between GlcA accumulation, GOX-1 secretion, and pH dynamics (acidification followed by recovery) further support GlcA-mediated pH homeostasis—a conserved fungal strategy to optimize enzyme activity [71, 72]. Further studies on *Ct*GlcAT are needed to elucidate its function and impact on *C. thermophilum*’s adaptability to diverse growth conditions.

## Conclusion

In this study, we demonstrate that *C. thermophilum* employs a non-cellobiose sensing mechanism to activate *Ct*Clr-2, driving co-transcription of the major cellulase CBH1-A and oxidative enzymes (LPMOs and CDH-1). The observed temporal secretion sequence—initial hydrolase secretion, followed by oxidases, and subsequent hydrolase release—extends the canonical oxidative priming model by revealing that early hydrolases generate requisite metabolic energy and reducing equivalents for LPMO activation. Critically, *Ct*Clr-2-mediated co-regulation of hydrolytic and oxidative enzymes, coupled with this temporal secretion program, ensures maximal cellulose degradation efficiency. These findings provide a molecular framework for engineering improved cellulolytic systems, with *Ct*Clr-2-driven co-regulation and staggered enzyme secretion dynamics guiding enzyme cocktail optimization, boosting the conversion efficiency of lignocellulosic feedstocks for biofuel and bioproduct synthesis. Such insights advance the development of sustainable biotechnological processes for renewable resource utilization.

## Supplementary Information

Supplementary material 1.

Supplementary material 2.

## Data Availability

Mass spectrometry proteomics data are available at ProteomeXchange/iProX (IPX0013993000). Transcriptomic data are deposited in NCBI SRA (BioProject: PRJNA1355141, PRJNA1355244). Omics analysis data and supplementary materials (figures, tables) are provided in Supplementary Table S5.xlsx and Supplementary Information.docx.

## Abbreviations

AA: Auxiliary activity
MCC: Microcrystalline cellulose
LPMO: Lytic polysaccharide monooxygenase
CDH: Cellobiose dehydrogenase
CBH: Cellobiohydrolase
BG: β-Glucosidase
EG: Endoglucanase
CDT: Cellodextrin transporter
CBT: Cellobionic acid transporter
CBAP: Cellobionic acid phosphorylase
GlcA: Gluconic acid
GLUT: Glucose transporter
GlcAT: Gluconic acid transporter
GMC oxidoreductase: Glucose-methanol-choline oxidoreductase
5'-UTR: 5'-Untranslated region
Glu-1P: D-glucose 1-phosphate
