# Supplementary material for: Integrated omics analysis of the cellulose co-degradation network of Chaetomium thermophilum
Source: Biotechnol Biofuels Bioprod. 2026 Jan 24;19:19. doi: 10.1186/s13068-026-02741-x (PMC12911045; doi:10.1186/s13068-026-02741-x)
Supplement: Supplementary file 1 — Supplementary material 1. [file 13068_2026_2741_MOESM1_ESM.docx]

**Supplementary Information**


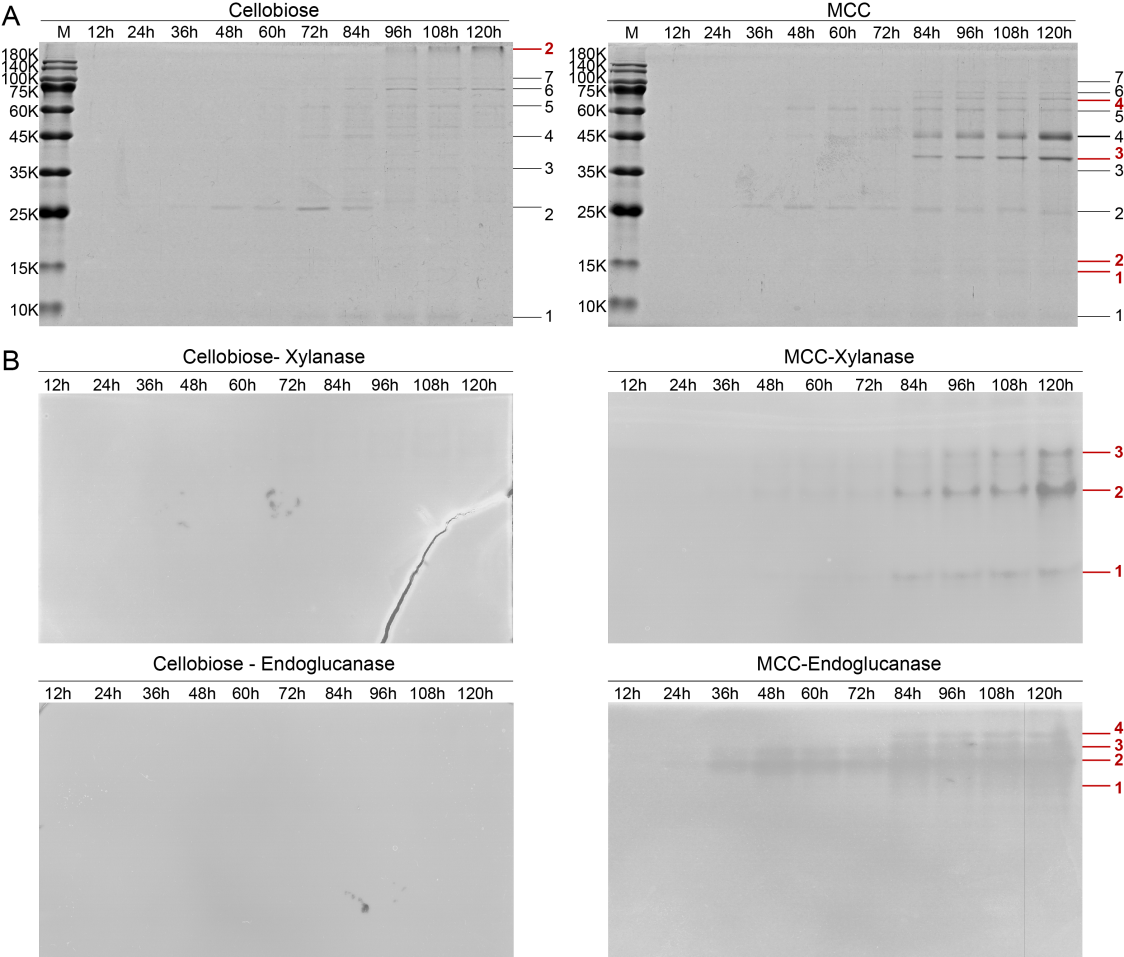


Fig. S1 Carbon source-dependent extracellular protein secretion and cellulase activities *in C. thermophilum*. (A) SDS-PAGE analysis of extracellular proteins secreted on cellobiose and MCC at 12–120 h. Samples were loaded at 10 μL per lane. Molecular weight marker (M) is indicated. Red marks denote substrate-specific induced bands, while black marks indicate shared protein bands. (B) Zymogram analysis of extracellular endoglucanase (substrate: CMC) and xylanase (substrate: xylan) activities. Samples were loaded at 10 μL per lane. Red marks denote substrate-specific induced activity bands, while black marks indicate shared activity bands.


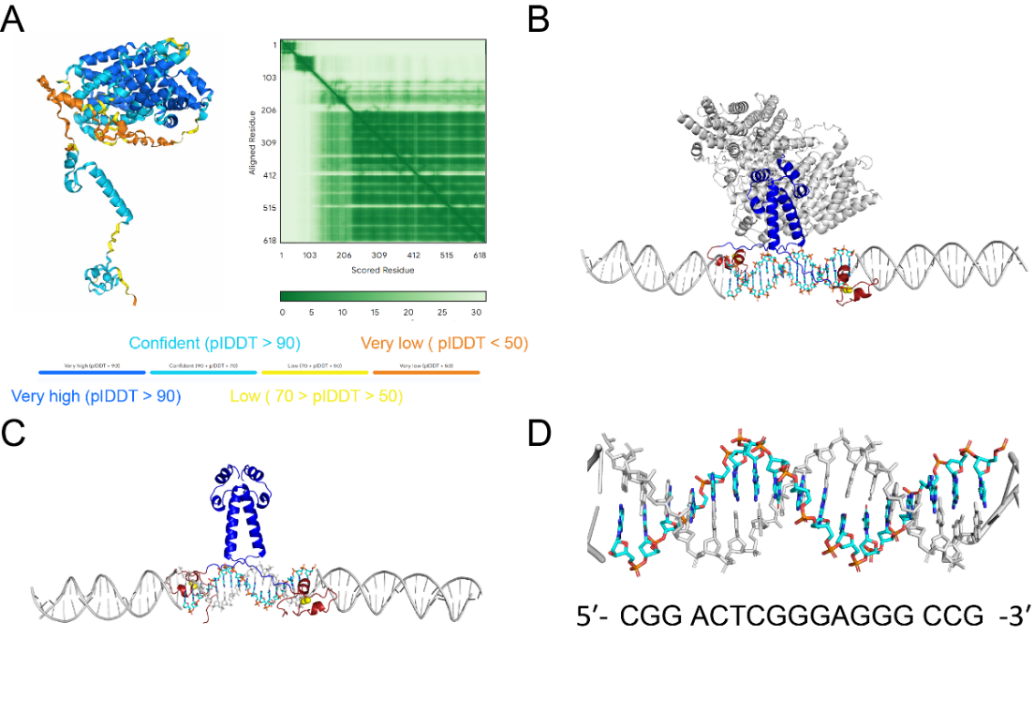


Fig. S2 AlphaFold 3 predicted *Ct*Clr-2 structure and its binding mode to the *cbh1-A* promoter region. (A) *Ct*Clr-2 structure. (B) *Ct*Clr-2 binds to the *cbh1-A* promoter region. (C) The binding mode of the Zn2Cys6-domain of *Ct*Clr-2. (D) *Ct*Clr-2 binds to the DNA sequence of the *cbh1-A* promoter.


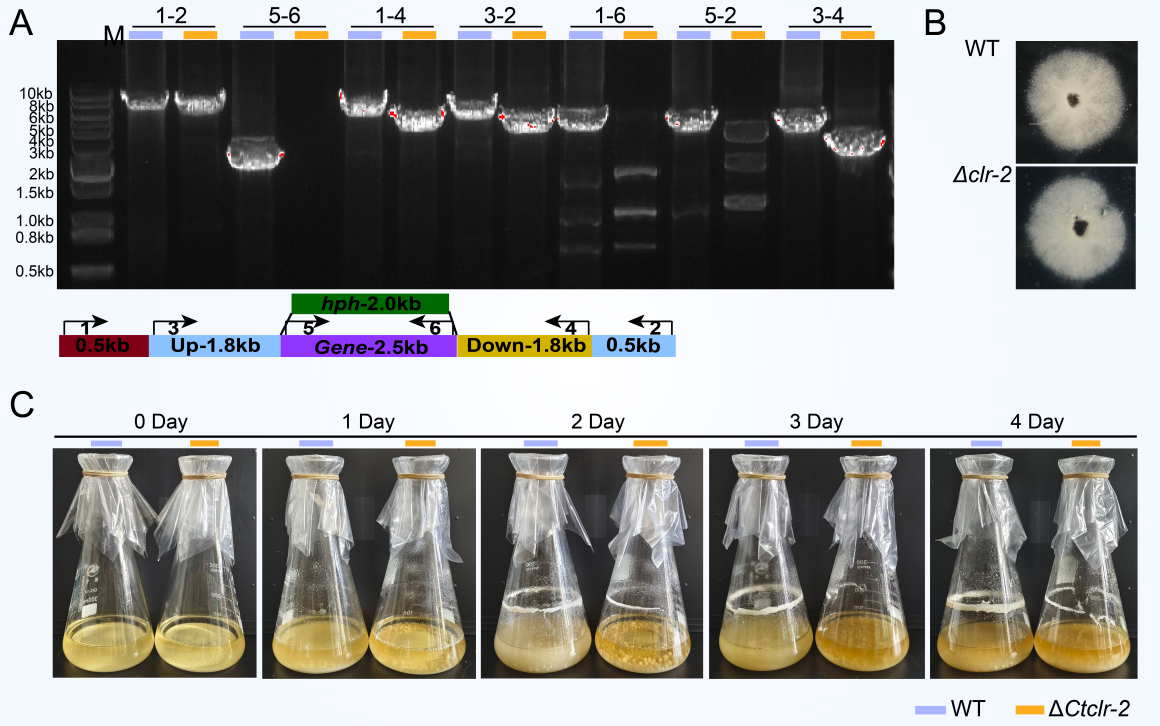


Fig. S3 Construction and phenotypic characterization of Δ*Ctclr-2* knockout strains. (A) PCR validation of *Ctclr-2* deletion via homologous recombination. Six primer pairs were used to distinguish WT (blue) and Δ*Ctclr-2*(orange) strains. Lane M: DNA molecular weight marker. The schematic depicts the gene structure and primer binding sites. (B) Growth phenotypes of WT (blue) and Δ*Ctclr-2* (orange) strains in MCC-containing medium over 4 days. Strains were cultured in liquid medium with MCC as the sole carbon source, and growth was monitored daily.


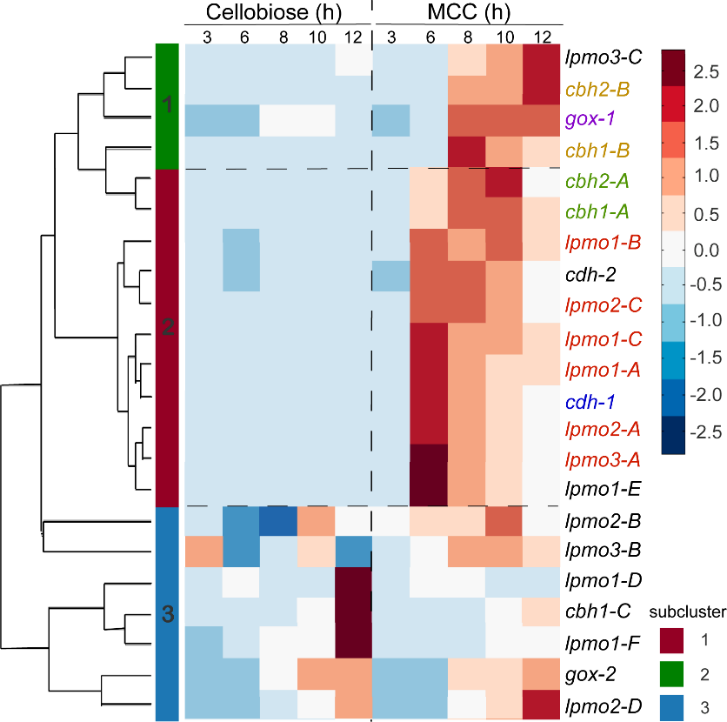


Fig. S4 RT-qPCR analysis of cellulose-degrading enzyme-encoding genes in *C. thermophilum* (2^-ΔCt^ values). The left color bar indicates enzyme subfamilies, and the right scale reflects relative transcript abundance.


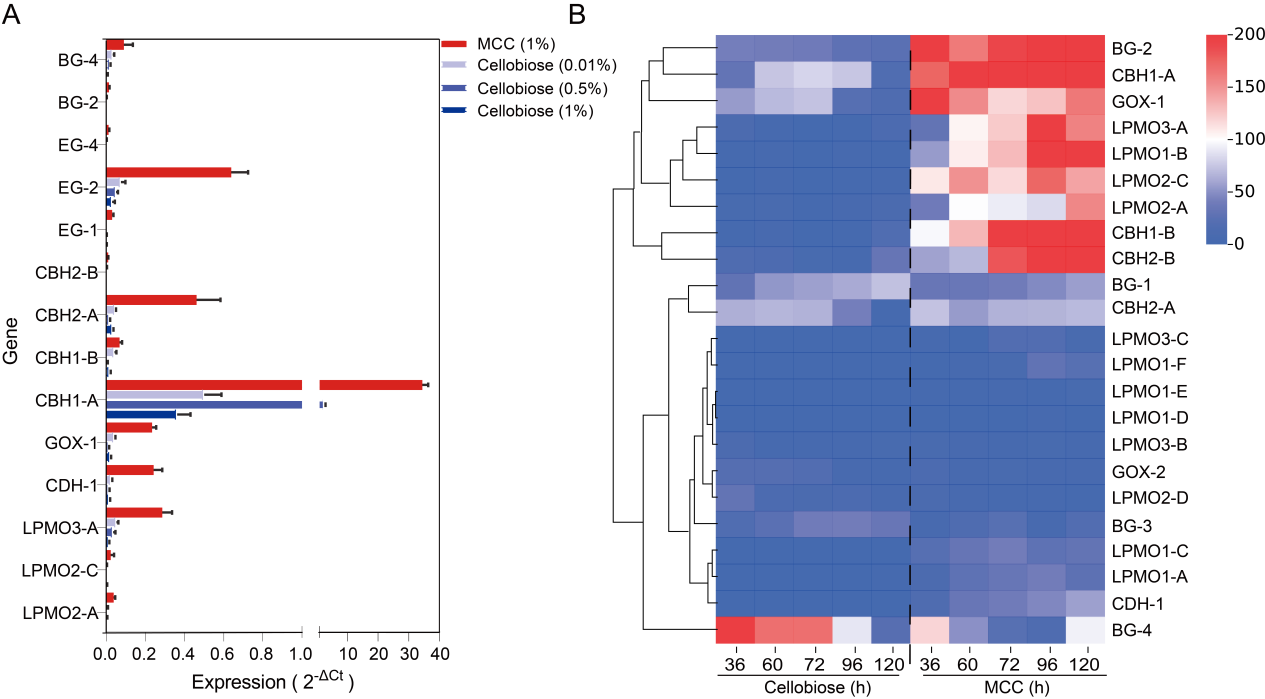


Fig. S5 (A) Concentration-dependent induction of cellulose degradation-related genes by cellobiose, with 1% MCC as a control. Cellobiose concentrations ranged from 0.01% to 1% (corresponding to reducing sugar levels of 0.1–10 mg/mL), and gene expression was quantified by qPCR (2^-ΔCt^ values). (B) Non-normalised hierarchical clustering heatmap of secreted cellulose-degrading hydrolases and oxidases in *C. thermophilum* cultured on cellobiose and MCC at 36, 60, 72, 96, and 120 h. The left colour bar indicates enzyme subfamilies, and the right scale reflects relative protein abundance.

**
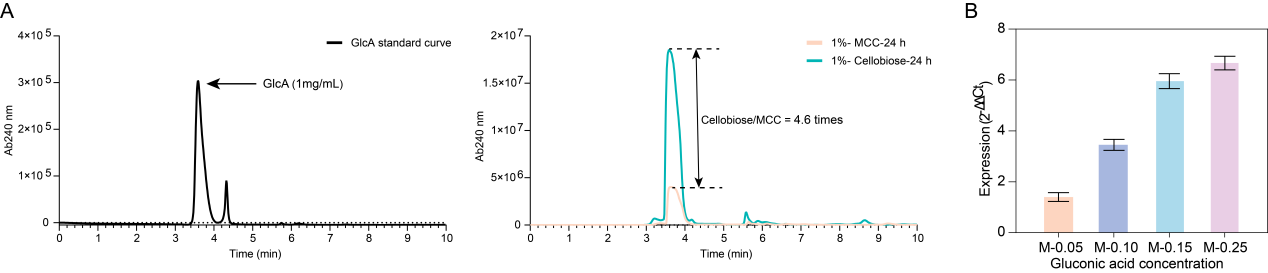
**

Fig. S6 (A) HPLC quantification of extracellular GlcA. Left: Standard curve for GlcA (1 mg/mL) detected at 210 nm. Right: Extracellular GlcA levels in cultures grown on 1% MCC or 1% cellobiose for 24 h. (B) Transcription of the GlcA transporter gene *CtglcAT* across GlcA concentrations (M-0.05 to M-0.25). Data are mean ± SD (n=3 biological replicates).

Table S1 Gene expression box construction primer

| Gene name | Primer name | Primer sequence |
| --- | --- | --- |
| *Ct*Clr-2 upstream sequence | Clr2-UF | GGATCTTCCAGAGATCGCCCTCTCATCTCGCTA |
|  | Clr2-UR | TTCAATATCAGTTAAGGTCGACATTGACGAGTATGCACCCC |
| Hygromycin gene sequence | Clr2-hphF | GGGGTGCATACTCGTCAATGTCGACCTTAACTGATATTGAA |
|  | Clr2-hphR | GTGTGATGATAGGCATGTGTGACAACCCAGGGCTGGTGACGG |
| *Ct*Clr-2 downstream sequence | Clr2-DF | CCGTCACCAGCCCTGGGTTGTCACACATGCCTATCATCACAC |
|  | Clr2-DR | CTGCCGTTCGACGATTCATGGAGGGGTTCATGACT |

Table S2 The primer of RT-qPCR

| Gene name | Uniprot | Primer | Primer sequence |
| --- | --- | --- | --- |
| *lpmo1-A* | G0RZR9 | TCCAGTCCTTAGGCATCCACAA | GATCGGTTTCCTTGAAGGCTCC |
| *lpmo1-B* | G0S408 | TGCGCAGCTGTATATGACGTG | GTTCATCGCCGAGTGGATGTT |
| *lpmo1-C* | G0RYQ6 | TCTCTGAAGTGGTCTAGCGAGG | ACGAAATGTAGAACTGGGCACC |
| *lpmo1-D* | G0S2P5 | TACCGACGTCAACTCTGACCA | GGCCGGGATGAGAAATGGATG |
| *lpmo1-E* | G0SB71 | CCCAGGGCCTCTCCATTTCTA | GATCTTGAACCACACGGGTCC |
| *lpmo1-F* | G0S0S9 | ATCCATGTTGACACGAATGGCA | GTCCCTTCCCTGCAAACTGAAA |
| *lpmo2-A* | G0RYX6 | GCACTACTTCGACATGCCACA | CGCACTCCATGTAGAACTGGG |
| *lpmo2-B* | G0SDM5 | AAAGTCTCCATCAACGGCCAAG | CTGACGTTGAGGATGGTCGATG |
| *lpmo2-C* | G0SH69 | ACAAGGGTCCTACCATGGCTTA | TGAATCTTGAACCAGCCGTCAC |
| *lpmo2-D* | G0S4L0 | TGTGACGTCGAGTCCTACTCC | TCCGTGTCGACTTGCAATGAG |
| *lpmo3-A* | G0S5P8 | AGGCCTATCCTCAGTGCATCAA | TCGTTGGCGGTATAGAGAGAGG |
| *lpmo3-B* | G0SA07 | ACCCCGATATCATCTGCCACA | CCAGGTGTCCCACTGGATGTA |
| *lpmo3-C* | G0S6H3 | GTGACGAGGTTACTGCCATCTG | TGAACTCTCCAGGGCACTTGTA |
| *cdh-1* | G0S434 | TACCTCAAGGACCCTAACGACC | GTGAGGTTGGCCACATTCTTGA |
| *cdh-2* | G0S3B0 | GAACTGGAGTGCCTTACGACTG | GCTCCTGCTGAGAAGATGACAC |
| *gox-1* | G0SG15 | CACTGGGATGGTATTGCTGACC | GGTTGTCGAACTGGACCTTGAG |
| *gox-2* | G0SAW6 | GGGCATTGTATGACAGCTCGAA | ATGATCTGCCGGTAGCTTTTGG |
| *cbh2-A* | G0SD43 | ACATGAACGTCCAGAAGTGCTC | TGGCAACGTGAGGAAGATTGAG |
| *cbh2-B* | G0S9B2 | AGCTCACAACCTCTACCCTCC | GAGAGGCCTGACGAGGCTTTA |
| *cbh1-A* | G0S477 | ACGAGTGGGATACCTCCATCTG | GTGACGAACTTGAGGTTCAGGG |
| *cbh1-B* | G0RYS9 | ACGCAGTTCTTCATCCAAGACG | AAGACCTTGAAGAGGTTGGTGC |
| *cbh1-C* | G0SBI7 | CAGACTTCGGTCGTCCTTGATG | AGGAGTTACCGGAGTAGCAGTC |
| *Ctcdt-1* | G0SC27 | TCGAGCGCGTCTAAGGAAAAC | GTACAGTTCGGCATTAtgcac |
| *Ctcdt-2* | G0SCI1 | ATGGGCATTCTCGATAAGAAGC | GTAGCGGAGGCAATGCAAAG |
| *CtglcAT* | G0SAI7 | ATGTTCTTCTCTCGTCGCAA | CTTGAAGTCGAGCTTGCCCT |

Table S3 Analysis of the *Ct*Clr-2 promoter binding site

| Gene | *Ct*Clr-1 | *Ct*Clr-2 |
| --- | --- | --- |
| *lpmo1-A* | N/A | CGG ACGGCTGCTGT CCG |
| *lpmo1-B* | N/A | CGG GGATGGGAATC CCG |
| *lpmo1-C* | N/A | CGG AGCCATTGTA CCG |
| *lpmo2-A* | N/A | CGG TGTTCAGCAGC CCG |
| *lpmo2-C* | N/A | CGG CTCTCACCACC CCG |
| *lpmo3-A* | N/A | CGG CGAAGGACCCC CCG |
| *cdh-1* | N/A | CGG CGAGCTTGCCG CCG |
| *cbh1-A* | N/A | CGG ACTCGGGAGGG CCG |
| *cbh2-A* | N/A | N/A |
| *cbh1-B* | N/A | CGG AGGGGGGGACT CCG |
| *cbh2-B* | N/A | CGG CCAAGCTCGTC CCG |
| *Ctcdt1* | N/A | N/A |
| *Ctcdt2* | N/A | N/A |
| *Ctclr-2* | N/A | N/A |

|  | Cellobiose sbustrate (PSMs) | | | | | Total | MCC sbustrate (PSMs) | | | | | Total |
| --- | --- | --- | --- | --- | --- | --- | --- | --- | --- | --- | --- | --- |
| Gene | 36 h | 60 h | 72 h | 96 h | 120 h |  | 36 h | 60 h | 72 h | 96 h | 120 h |  |
| LPMO1-A | 0 | 0 | 0 | 0 | 1 | 1 | 11 | 28 | 33 | 40 | 26 | 138 |
| LPMO1-B | 1 | 4 | 0 | 4 | 1 | 10 | 55 | 108 | 131 | 224 | 223 | 741 |
| LPMO1-C | 0 | 0 | 0 | 0 | 0 | 0 | 22 | 32 | 40 | 28 | 30 | 152 |
| LPMO1-D | 0 | 0 | 0 | 0 | 0 | 0 | 0 | 0 | 0 | 3 | 0 | 3 |
| LPMO1-E | 0 | 0 | 0 | 0 | 0 | 0 | 2 | 1 | 3 | 2 | 6 | 14 |
| LPMO1-F | 0 | 0 | 0 | 0 | 0 | 0 | 0 | 4 | 8 | 29 | 23 | 64 |
| LPMO2-A | 6 | 8 | 1 | 5 | 9 | 29 | 38 | 99 | 92 | 84 | 156 | 469 |
| LPMO2-B | 0 | 0 | 0 | 0 | 0 | 0 | 0 | 0 | 0 | 0 | 0 | 0 |
| LPMO2-C | 0 | 3 | 0 | 2 | 3 | 8 | 110 | 151 | 117 | 174 | 142 | 694 |
| LPMO2-D | 30 | 12 | 9 | 12 | 5 | 68 | 9 | 5 | 3 | 3 | 1 | 21 |
| LPMO3-A | 11 | 4 | 0 | 1 | 12 | 28 | 30 | 105 | 124 | 216 | 159 | 634 |
| LPMO3-B | 10 | 3 | 4 | 4 | 6 | 27 | 8 | 5 | 4 | 6 | 3 | 26 |
| LPMO3-C | 0 | 0 | 0 | 0 | 0 | 0 | 3 | 0 | 19 | 19 | 8 | 49 |

Table S4 Extracellular secretion levels of LPMOs in*C. thermophilum* quantified via proteomics

Table S6 Sequence similarity analysis

| Gene name | Gene ID | Organism | Sequence | | | Gene description |
| --- | --- | --- | --- | --- | --- | --- |
|  |  |  | Identity (%) | Cover (%) | Total score |  |
| *Ct*CDT-1 | CTHT_0055680 | *Chaetomium thermophilum* | 74.4 | 96.5 | 2099 | Sugar (and other) transporter |
| CDT-1 | NCU00801 | *Neurospora crassa* |  |  |  | cellodextrin transport-1 |
| *Ct*CDT-2 | CTHT_0057310 | *Chaetomium thermophilum* | 78.5 | 94.5 | 2079 | Sugar (and other) transporter |
| CDT-2 | NCU08114 | *Neurospora crassa* |  |  |  | cellodextrin transport-2 |
| *Ct*CBT | CTHT-0047580 | *Chaetomium thermophilum* | 79.1 | 96.1 | 2030 | Sugar (and other) transporter |
| CBT | NCU05853 | *Neurospora crassa* |  |  |  | Cellobionic acid transporter |
| *Ct*CBAP | CTHT-0019620 | *Chaetomium thermophilum* | 80.0 | 97.7 | 2513 | Uncharacterized protein |
| CBAP | NCU09425 | *Neurospora crassa* |  |  |  | cellobionic acid |
